# Learning mathematics in two dimensions: a review and look ahead at teaching and learning early childhood mathematics with children’s literature

**DOI:** 10.3389/fpsyg.2014.00459

**Published:** 2014-05-20

**Authors:** Lucia M. Flevares, Jamie R. Schiff

**Affiliations:** Department of Teaching and Learning, The Ohio State UniversityColumbus, OH, USA

**Keywords:** picture books, children’s literature, early childhood, mathematics, geometry, professional development

## Abstract

In the past 25 years an identifiable interest in using children’s literature in mathematics learning emerged (Clyne and Griffiths, 1991; Welchman-Tischler, 1992; Hong, 1996; Hellwig etal., 2000; Haury, 2001). We critically review the rationales given for the use of picture books in mathematics learning, with a special focus on geometry due to its underrepresentation in this body of literature and the need for greater focus on this topic. The benefits and effectiveness of using picture books for children’s mathematics learning and interest have been documented (Hong, 1996; O’Neill etal., 2004; Young-Loveridge, 2004). For geometry, although much learning of shape ideas should be hands-on, two-dimensional figures are essential to develop children’s understanding of plane geometry. Books may effectively engage pre-literate children with plane shapes (van den Heuvel-Panhuizen and van den Boogaard, 2008; Skoumpourdi and Mpakopoulou, 2011) and shapes as gestalt wholes or prototypes (van Hiele, 1986; Clements etal., 1999; Hannibal, 1999). We review several guidelines and evaluative criteria for book selection, including Cianciolo (2000), Schiro (1997), Hunsader (2004), and van den Heuvel-Panhuizen and Elia (2012). Geometry concepts have proven challenging for young students, but their difficulties may stem, in part, from inadequate teacher training and professional development (Clements and Sarama, 2000; Chard etal., 2008) which lead to misconceptions (Oberdorf and Taylor-Cox, 1999; Inan and Dogan-Temur, 2010). Using picture books in teacher training may be an inviting way for early childhood teachers to enhance their own knowledge. We will examine the literature for guidance on incorporating children’s literature into teacher training. In closing we will outline a comprehensive, multi-pronged agenda for best instructional practices for selection and use of children’s books in mathematics activities and for teacher training.

When children see a storybook or a picture book, they may not immediately associate it with mathematical ideas, but their teachers and scholars within the early childhood education and mathematics education communities have increasingly recognized the potential for using storybooks and picture books to aid in children’s mathematics learning. However, the selection, evaluation processes, and implementations of these books for learning are not trivial processes and have become the subject of much writing and research.

Within this review we endeavor to shine light on the rationales and goals for using children’s literature in mathematics learning and on the competing factors and viewpoints that influence this pursuit such as the push and pull between deep analysis and ease of use when evaluating books. Our review below begins with a brief chronology of literature on using children’s literature in mathematics learning. Then we present the rationales for using children’s literature in mathematics learning including developing the children’s with regard to the National Council for Teachers of Mathematics (1989) mathematical processes, and their motivation and cognitive engagement. Throughout we identify and discuss the themes of scholarship of and advice for practicing teachers to choose and use children’s literature effectively in mathematics. Through examples and within the section on rationales we devote a particular focus on using children’s literature for geometry learning due to a significant gap in focus from both research and teacher professional development.

In the next section we address picture book evaluation processes for use in mathematics learning. In doing so, we especially attend to the selection of evaluation schemes relative to the purpose. We next present a review of professional development to support teachers’ use of children’s literature, with an eye toward early childhood geometry in particular. Lastly, we present recommendations for both research and practice, aimed at framing the research-practice cycle going forward.

For this review we refer first to van den Heuvel-Panhuizen and Elia’s (2012) comment, “By “picturebooks,” we mean books typically containing text and pictures in which pictures have an essential role in full communication and understanding (Nikolajeva and Scott, 2000). Arizpe and Styles (2003) stressed that a picture book is a “book in which the story depends on the interaction between written text and image and where both have been created with a conscious esthetic intention” (p. 18). However, we note that other, broader, definitions may be considered, such as, “*mathematics literature* references any piece that has the potential to engage children in mathematical conversations” (Nesmith and Cooper, 2010, p. 280). As such, we would amend the definition to include wordless photography books or illustrated books, which may provide engaging, thought-provoking visuals for discussion and activities (e.g., Whitin and Wilde, 1995; Moyer, 2000). Additionally, even in the primary grades some books may be productive choices for engaging children with mathematical ideas even though they have few, if any pictures. While such variability in what may be considered children’s literature for mathematics learning creates a complication for synthesizing across published works, using a broad definition of children’s literature for mathematics learning reflects the topic as addressed today.

## CHRONOLOGY OF CHILDREN’S LITERATURE IN MATHEMATICS LEARNING

Now a popular topic, the idea of using books as media for children’s learning originated long ago: van den Heuvel-Panhuizen and Elia (2013) may have identified the genesis of the idea of young children learning through books, especially picture books, back to 1652 with Comenius’ *Orbis Pictus.* However, while Comenius’s idea provides an origin for using illustrated books for children’s learning, concerted attention to their use for mathematics learning is a far more modern phenomenon.

Within more modern education literature, the topic of using children’s book for mathematics learning began to emerge with initial direction toward the topic such as lists of suggested books from Beard (1962), Whitaker (1962), and Bravo (1965). None of these brief articles articulated the pedagogical processes in detail and for a couple of decades very little was written about this practice.

A notable departure from those works in text and symbol processing in mathematics learning came with Farr, 1979 article aimed at school librarians. She argued that with the adoption of socioconstructivist views of learning in primary grades, instruction needed to include both concrete hands-on experiences for mathematics learning and materials such children’s books that could stimulate interactions. Unsurprisingly, given the lack of prior scholarly focus, she noted the dearth of appropriate trade books and found that those existing at the time often took a historical or instructional perspective and would not engage young learners. She called for books for mathematics learning that would be appealing, contain accurate information with precise use of any terms and clear presentation of concepts.

Such a well-reasoned call in a widely circulated school journal likely did contribute to the increase in trade books related to mathematical ideas for children, but much more remained to be delineated regarding the use of these books. In response to Farr (1979), Radebaugh (1981) offered lists of books for each of several early childhood mathematics topics, and a rationale for using books and suggestions for implementation. With these suggestions Radebaugh offered persuasive, practical advice such as using shape pictures in books as the basis for exploring hands-on shapes, although the work lacked evidence of the effectiveness of the practice of integrating children’s books into mathematics learning. Likewise, Harsh (1987) delved into two popular children’s picture books for their good fit with hands-on concrete activities to promote pre-number understandings but offered no empirical evidence of their effectiveness. Here and throughout this review, we recognize the value in practical advice for teachers, often from fellow teachers, but to propel the understanding of children’s literature for mathematics learning forward, we also acknowledge the role of research to document relevant variables for successful implementation.

Since the early 1990s, a steady increase in the number of publications on the integration of children’s literature into mathematics can be found (e.g., Clyne and Griffiths, 1991; Welchman-Tischler, 1992; Whitin, 1992; Haury, 2001), typically aimed at offering resources to teachers. This uptick of publications on the topic did not come out of the blue. As cited by Marston (2010) and van den Heuvel-Panhuizen (2012), the publication of the first Standards from the U.S. National Council of Teachers of Mathematics National Council for Teachers of Mathematics (1989) propelled interest in integrating children’s literature into mathematics experiences. California, an influential force in U.S. educational practices with its large and diverse population, developed criteria for books to be included in the state mathematics program (Donoghue, 1996), and trade-book lists accompanied six of the 12 textbook series acceptable for adoption in California public schools. The book lists classified a book as acceptable for one or more of 12 mathematics topics common to the early grades. These events spurred the publication of trade books expressly intended for mathematics learning and of the study of such books and their implementation. Later *Standards* from the U.S. National Council of Teachers of Mathematics (2000) reinforced the use of children’s literature for mathematics learning, but Marston characterized the resulting wave of new books as being of dubious literary quality. Thus, with such high-profile endorsements, the support for using children’s literature for mathematics learning had spread but not necessarily in a consistent and high-quality manner.

Within those publications some have had the explicit goal of documenting how children’s literature can have a positive outcome for mathematics learning (Jennings et al., 1992; Hong, 1996; Young-Loveridge, 2004) and provided evidence that the use of picture books in the early years of schooling can also contribute to the learning of mathematics. As the study of the topic has evolved, important aspects have been addressed, although each time prompting additional questions such as about the distinct contribution offered by the literature within the ongoing mathematics learning (Young-Loveridge, 2004) or the robustness of the results (Jennings et al., 1992). Some of this work will be discussed in greater detail below. Many of the publications about using children’s literature in mathematics have been practitioner focused, presenting narratives from their classrooms of using literature for mathematics learning and/or presenting lesson activity examples and ideas, offering the realization of prior research or theory-based work.

Through its rise in popularity, the integration of children’s literature with mathematics learning has grown to encompass many methodologies, foci and interest around the globe. The development of this topic has also been international, with the most prominent lines of scholarship coming from North America, Europe, and Australia. More recently, the study of the integration of children’s literature into mathematics learning has included more rigorous scholarship, aimed at providing evidence of the efficacy of books in mathematics learning and teaching. Simply selecting and using a book with mathematics learning experiences, analogous to providing hands-on materials, does not guarantee learning. From the history of work on using children’s literature for mathematics learning, we now examine this work more closely, focusing on the rationales for using the books and the evidence for their use.

## RATIONALE FOR USING CHILDREN’S BOOKS

As the focus on children’s literature as a resource and tool for mathematics learning became more prominent, authors identified additional rationales for using these books and functions of these books. Rather than borrowing one of the lists of reasons (e.g., Schiro, 1997) we recognize some overlap of rationales and purposes in which, for instance, while one article may name a rationale of nurturing children’s positive dispositions toward mathematics, another cites the motivational potential of using picture books. Thus, we have codified the rationales into categories reflecting both emphases within the articles and their importance within standards and the larger body of literature.

Additionally, some of the rationales found in the extant literature encapsulate procedures such as to introduce manipulatives, to prepare students for a mathematics skill or concept (Price and Lennon, 2009) or to review a concept. Phrasing as such may imply a limited role for the book, not to provoke problem solving or analysis or deep engagement with the mathematics and the book. We will not review articles or elements of articles that focused on narrow procedural or functional aspects which an interested reader may find in practitioner-focused journals. Thus, rather than advice on procedural implementation, we focus here on deeper, more ambitious rationales for using children’s books for mathematics learning. Even still, these rationales may overlap and are not mutually exclusive. For instance, making connections between a book’s illustrations and hands-on experiences could also relate to the support children’s representational understandings.

We first discuss each of the mathematical processes, socioemotional mechanisms, and the rationale and goal of reaching all learners that have been endorsed for using picture books. Then we pay particular attention to the use of children’s literature to support young children’s learning of basic geometric and spatial concepts.

Not all, and perhaps not many, articles addressing the integration of children’s literature for mathematics learning have provided empirical evidence of the efficacy of this practice. Where authors have provided empirical evidence for effectiveness, we discuss that in turn and we identify the most significant gaps for additional empirical evidence to address in the following sections and in the closing section.

### MATHEMATICAL PROCESSES

First, picture books could be seen as a means for developing young learners’ understandings of the five mathematical processes standards of communication, representation, connections, problem solving, and reasoning and proof (National Council for Teachers of Mathematics, 1989). We also note below, where relevant, where these processes may overlap with the recent Mathematical Practices from the Common Core State Standards (National Governors Association Center for Best Practices, Council of Chief School Officers, 2010), currently being adopted in most of the United States.

### COMMUNICATION

Taking a Vygotskian, sociocultural perspective, some authors have highlighted the social aspect of whole-class or small-group book reading as a site for interaction and sharing of ideas presented by or analyzed through the illustrations and text. Bringing shared literature in mathematics engages and socializes children into shared reading and learning; the books can also be a springboard for mathematical discourse between children and adults both at school and at home. Anderson et al. (2004, 2005) observed parent-child dyads in book readings and documented the shared mathematical discourse during the reading and parents’ scaffolding of children’s thinking. They presented fine-grained discourse analyses well, identifying how parents supported children’s ideas, but they also stressed that story reading at home typically differs from book reading in classrooms. Their work raised important questions for how home-school connections could support literature for mathematics learning. Their work underscored the need for the educational community to offer support to families regarding book choices and perhaps also guidelines for at-home reading. We also note a potential particular needed for geometric concepts that may be less familiar than number-related concepts.

In the classroom, teachers can serve a facilitative role in the discourse around picture books, but Elia et al. (2010) and van den Heuvel-Panhuizen and van den Boogaard (2008) persuasively argued, with accompanying discourse analyses of children discussing the measurement and spatial concepts in books, that the children’s self-initiated mathematics talk itself deserves study. The picture books they used engaged the children in reflection on mathematical concepts as evidenced by the children’s spontaneous utterances during picture book reading. Here, again, not all books are equal in quality or fit for a group of students or a particular learning goal. These researchers, like Anderson et al. (2004), found that children’s utterances differed in amount and kind including problem-solving statements based on the picture book used. However, looking forward, individual and situational variables must be considered to support learner’s mathematics discourse.

Additionally, using children’s literature has been deemed supportive of introducing vocabulary (Griffiths and Clyne, 1988; Doig, 1989; Welchman-Tischler, 1992; Charlesworth, 2005). However, the necessary characteristics of the books involved and the means by which this vocabulary development may be achieved have not been well explicated. Some good empirical evidence came from Jennings et al. (1992) intervention study in which children in the literature-condition performed better on vocabulary assessments, as well as achievement and interest indices, following the 5 months intervention than children in the control kindergarten class. Their study is now over two decades old and more work is still needed to document how to support communication processes including mathematics vocabulary learning and mathematically rich discourse through experiences with story and picture books. Finally, with the outlining of the mathematical practices from the Common Core State Standards (National Governors Association Center for Best Practices, Council of Chief School Officers, 2010) future work should address how literature may aid young learners in going beyond simple communication to the third practice, “Construct viable arguments and critique the reasoning of others.”

### REPRESENTATION

Closely intertwined with mathematical communication, the process of representation stems from the natural connection between words and images. Focusing on this link, van Oers (2013) acknowledged the potential benefits of children’s books for supporting discourse, especially when the discourse can be propelled by illustrations that support children’s thinking about mathematical ideas.

Illustrations within picture books may be seen as representations of and for mathematical ideas for learners, to be recognized by them or scaffolded for recognition by their teachers, van den Heuvel-Panhuizen et al. (2009) asserted that, the text and illustrated representations may serve as cognitive hooks for young learners (Lovitt and Clarke, 1992) through the conduit provided from the book to other familiar experiences. The vignettes from their observations documented children’s uptake of book illustrations as representations to engage in mathematical cognition. However, we must again remind readers of the caveat of *the potential* for a book or even only the *potential of some books,* and not inherent qualities for all books designated as for mathematics learning. Some books with intentional mathematical content have illustrations that are representationally poor in terms of the mathematical concepts, such as a shape labeled a square but with sides that are not straight lines or four sides but not all equal in length.

Additionally, even having attractive, engaging, mathematically relevant illustrations and representations is not enough. The correspondence between text and illustrations must be coherent. As Farr (1979) warned that discrepancies between a book’s illustrations and the accompanying text can cause confusion, especially for the topics of time, distance, and spatial relations. Whether a book has text or is a book solely of photographs or illustrations, the mathematical concepts it purports to represent should be accurate representations of those concepts. Beyond accuracy, picture books’ representations vary in their richness and presentation of exemplars. For example, a picture book may show triangles of various colors and sizes, but if they are all equilateral triangles, children experience a narrow representation of a triangle. In contrast, another picture book may include only a page or two with illustrations of triangles but include several scalene, isosceles, and right triangles as well as equilateral. Insufficient attention has been paid to the pictures within picture books as representations; many books may be pleasurable reading experiences but problematic for supporting learners’ mathematical representations (e.g., Marston, 2010).

### CONNECTIONS

Illustrated books may have a unique potential to promote young learners’ connections between mathematical ideas and their experiences even out of school (Moyer, 2000). The union of text and pictures provided by books can support connection-making for learners at multiple levels as described by the National Council for Teachers of Mathematics (1989): among mathematical ideas and in contexts outsides of mathematics, to other content areas and to real-world or personal experiences (van den Heuvel-Panhuizen and Elia, 2012).

Often the text and pictures provide the topic, the anchor for mathematical discussions, from two-dimensional book pages to activities, typically with concrete representations (Anderson et al., 2004), and connections can bloom from there. From their classroom experiences, Shatzer (2008) and Hellwig et al. (2000), for example, highlighted the potential for well-selected literature to both support mathematical concept formation and bridge from the mathematical concept to students’ lives. Connections may emerge spontaneously as students engage with a book individually or in a group, but when intentional, they must be authentic and supported. Hellwig et al. (2000) and Nesmith and Cooper (2010) pressed for connections to be meaningful and authentic to the children experiencing the books and for the connections to be as a web of interrelated ideas, rather than isolated. Given potentially significant differences by age, prior experience, and other learner characteristics, teachers, parents, and other stakeholders would benefit from more guidance for finding and using books that would connect meaningfully to a classroom or individual young learner.

### PROBLEM SOLVING

Although a book reading activity can be its own engaging forum for mathematical thinking, books are frequently used as prior to hands-on activities, with the relevant concepts bridging the reading and hands-on activities. Skoumpourdi and Mpakopoulou (2011) offered good evidence and an effective rationale for using children’s literature “to provide a model, illustrate a concept, pose a problem and stimulate an investigation” (p. 199), by using informal knowledge and experiences to connect to more formal mathematical problem solving.

Storytelling, engaging in narratives, in mathematics has been promoted by Zazkis and Liljedahl (2009), O’Neill et al. (2004), and Casey et al. (2004). This subset of work has outlined how engaging learners in storytelling can spark students’ interest, engage them in mathematical cognition, reduce anxiety, and support the building of positive relationships between teachers and students and student peers. Story characters can be especially engaging means for posing problems (Casey et al., 2004; Skoumpourdi and Mpakopoulou, 2011) as children make sense of problem situations through the character’s situation within the book, experiencing CCSS Mathematical Practice #1, “Make sense of problems and persevere in solving them.” Problem solving using picture books is also endorsed through a modeling approach, a vigorous line of research in mathematics education, can effectively include children’s literature in modeling activities, to pose a problem, to provide information, to offer a context, to provide compelling characters in the posing of problems for modeling (English, 2010; Flevares and Schiff, 2013). For example, by following the actions of a dog named Baxter, students in English’s study sorted and classified items and used them for data analysis in modeling a problem solution. The story offered an effective context for the children’s problem solving.

### REASONING AND PROOF

Lastly, the extant research and practitioner-focused articles on using literature for mathematics learning lack attention to the process of reasoning and proof. Only Marston et al. (2013) specifically addressed supporting students’ reasoning, although others may have intended or assumed the process as embedded within problem solving activities. Marston, Muir and Livy discussed the implementation of a few picture books and how the books facilitated grade 1 and grade 2 children’s engagement in concepts including through problem posing and generating and discussing multiple solutions. These activities entail reasoning, but yet, the process of reasoning could yet be more specifically addressed, particularly for the book’s role in supporting the children’s reasoning. Supporting children’s reasoning from the givens within a book’s text and with the representations on a two-dimension book page merits further study.

## EMOTIONAL AND MOTIVATIONAL ASPECTS

Some of the attractiveness of using children’s literature stems not from its support for content learning and cognitive engagement but their potential for children’s social and emotional growth (Hong, 1996), encouraging the learners’ persistence and goal-related, motivational behaviors (Ray and Smith, 2010). The motivational potential of children’s books for engaging children in mathematics learning was also cited by Griffiths and Clyne (1991), Murphy (2000), Usnick and McCarthy (1998), and van den Heuvel-Panhuizen and van den Boogaard (2008), who noted how books can elicit emotional connections in learners, engaging them on multiple levels. One picture book often cited for learning primary grades shape concepts is *The Greedy Triangle* (Burns, 1994), which while presenting shapes in the plot, concludes with a moral of being happy with who you are. Well-selected books can successfully serve these multiple functions.

Hong (1996) cited the catalytic motivational property of children’s literature for children’s engagement with mathematics. Her own work provided evidence of storybooks’ positive effects on children’s dispositions toward mathematics learning as she documented students’ increased preference for mathematics following the intervention with picture books. Thus, books may have the potential to offer an inviting, motivating context for mathematics learning. Experiences with picture books may spark children’s curiosity, for instance, about fundamental geometric concepts as design and build with blocks in their classrooms.

### REACHING ALL LEARNERS

Storybooks, if appropriately chosen, have the potential for supporting the mathematics learning of students of a wide range of learner characteristics including students with disabilities (Courtade et al., 2013) and students with low self-efficacy for learning mathematics (Jenner, 2002). Book reading may support positive cultural identity formation for students with regard to mathematics learning (Edelman, 2012), but care must be taken to select literature appropriate to a student population (Hefflin and Barksdale-Ladd, 2001), regardless of the academic content.

van den Heuvel-Panhuizen (2012) documented how interventions with picture book reading can be such broadly worthwhile and meaningful activities that they reach and support children of a wide range of background characteristics. She noted especially that picture books have been effective for supporting the learning of those with home languages different from those of the classroom or those from lower socioeconomic backgrounds and for girls. Casey et al. (2008) have documented gender-related effects of stories on mathematics learning, with regard to spatial concepts. Their hands-on intervention with stories engaged the children with puppets, chants, and movement. Based on their evidence, girls especially may benefit from an explicit, systematic approach to spatial reasoning experiences in classroom settings, making up the gap from what boys pick up otherwise. The numerous potential positive effects of using children’s literature in mathematics learning await being more effectively harnessed as further delineation is needed identifying and guiding the selection and use of books.

### CHILDREN’S LITERATURE FOR GEOMETRY LEARNING

As acknowledged by Clements et al. (1999) number-related topics dominate curricula and standards during early childhood and the primary and elementary grades. This predominance may have compromised the development of children’s geometric and spatial concepts. The National Council of Teachers of Mathematics (2000) recommended a more equal balance between number concepts and geometry concepts, and for the United Kingdom, Jones and Mooney (2003) warned of the significant underrepresentation of geometry within the National Curriculum and the National Numeracy Strategy. We concur and argue that supporting learning of geometric concepts and spatial reasoning should be a priority moving forward for the study of children’s literature in mathematics learning. Children’s literature can play an important role in this mission and we now turn to examine the role of books in geometry concept learning especially.

Although less attention has been paid to story and picture books with geometric and spatial ideas than those with numerical topics, we argue that these books may be especially effective tools for these concepts. For geometry, although much learning of shape ideas should be hands-on, two-dimensional figures are essential to develop children’s understanding of plane geometry. Books can effectively engage pre-literate children with plane shapes (van den Heuvel-Panhuizen and van den Boogaard, 2008; Skoumpourdi and Mpakopoulou, 2011) and shapes as gestalt wholes or prototypes (van Hiele, 1986; Clements et al., 1999; Hannibal, 1999). Although Hannibal focused on shape learning and not particularly books for that shape learning, an important connection may be extracted: books can become grounds for presenting or reinforcing misconceptions about shape. She wrote that “perfect shapes are referred to as the best example or the prototypical triangle or rectangle that is most frequently presented in shape books, posters, puzzles, and toys. Therefore, for example, children would frequently not recognize a scalene triangle as a triangle because ‘it is too crooked”’ (p. 354). As of a result of incorrect, excessively narrow definitions and defining experiences, a child may develop a geometric misconception such that a square is not a rectangle by age five (Clements et al., 1999). Unfortunately, many picture books with shapes labeled may reinforce or encourage this misconception with different pages for “squares” and “rectangles,” labeled as such, implying they are non-overlapping categories, and with no squares shown on the pages designated as “rectangles.” Instead, high-quality literature could provide mathematically correct representations of shapes and related geometry concepts.

In both the vernacular and mathematical senses of the word, images within books can serve as “representations,” and thus, picture books featuring shapes, whether illustrations or photographs, can be valuable stimuli for presenting basic geometric shapes in two-dimensional form. These two-dimensional representations can then be recognized and identified within the three-dimensional environment. The classroom teachers in Minetola et al. (2012) study conducted a geometry learning experience that fostered connections between shapes in their environment and items new to them, using Hoban’s photography books, *Shapes, Shapes, Shapes* (Hoban, 1986), and *Cubes, Cones, Cylinders, and Spheres* (Hoban, 2000). The photographs within these books are not simple, isolated plane shapes, but rather shapes within visually complex photographs of people, places, and things. The authors focused well on connections and mechanisms for learning, noting that the children transferred their recognition of the two-dimensional shapes from the pictures to activities in their three-dimensional environment. Hands-on activities such as block building and tile designs are the three-dimensional tactile, haptic experiences with perception and depth. Paired with books representing shapes and spatial relationships, children can engage in seeing shapes in a two-dimensional plane and translating them to three-dimensional space, making conceptually meaningful connections.

Shape and spatial representations in books can provide provocative shared referents for small-group or large-group discourses when well implemented by teachers. From their research into the efficacy of using picture books, van den Heuvel-Panhuizen et al. (2009) provided powerful examples of visualization and discourse in the small-group discussions with the read-alouds by the teacher. These read-alouds and discussions promoted children’s thinking regarding spatial relations in their familiar environment. The authors made a logical argument and provide good evidence for how experiences with books can thus support children’s understanding of the geometric-spatial features of their familiar environment. To extend this work further, we can look to van den Heuvel-Panhuizen and Elia’s (2012) framework for evaluating picture books. They distinguished three areas of geometry: orienting, regarding relative spatial sense; constructing, regarding composition in two- and three-dimensions; and operating with shapes and figures. These categories, in addition to basic shape recognition and shape attributes, will be productive for future studies of the research into learning experiences with picture books, including identifying books compatible with each of these categories for the breadth of geometry concepts. In the next section we explore their evaluation scheme and others for the features, including mathematical content evaluated for use in children’s learning.

## PICTURE BOOK EVALUATIONS

In this section we present and compare the criteria that have been developed for evaluating children’s literature for mathematics learning. We highlight differences and commonalities in the criteria, foci and goals of the rubrics and recommendations. Later we discuss recommendations for applying these criteria to books and on the evaluation processes for teachers.

Educators should expect guidance from research on selecting and using children’s literature in their mathematics teaching to find books that will both engage the young learners. The need for identifying and using only high-quality books cannot be overestimated (Whitin, 2002; Nesmith and Cooper, 2010). Rather than simply ineffectual, real dangers for learning may come from incorporating low-quality books in learning experiences. Didactically written “storybooks,” which Nesmith and Cooper referred to as “pseudotextbooks,” can be ineffective, unengaging, de-motivational, potentially harming students’ interest in mathematics. A low-quality book used with the intent of mathematics learning may suggest or reinforce the mathematics is inherently uninteresting and requires a book accompaniment for engaging the children (Whitin, 2002), essentially presenting the book as a spoonful of sugar with the implied unpalatable medicine of mathematics.

To review the various book evaluation criteria, we looked for defined evaluation or selection criteria for systematic assessments of books for mathematics learning, as opposed to principles or more general guidance. Few sources met this criterion, as noted by van den Heuvel-Panhuizen and Elia (2012). We will focus on evaluation schemes devised or refined by Schiro (1997), Hellwig et al. (2000), Hunsader (2004), Nesmith and Cooper (2010), and finally van den Heuvel-Panhuizen and Elia (2012).

The formal efforts to evaluate children’s literature for mathematics learning can largely be traced back to Schiro (1997) who created standards addressing both the mathematical and literary quality of books. He stated that began the process of creating literary and mathematical evaluation standards for excellence in 1990 with the aim of filling a perceived gap – no standards existed at the time. Schiro drafted exhaustive criteria, resulting in a 14-page evaluation instrument. In addition to the attributes of mathematical literary criticism he outlined, his mathematical standards included criteria for, mathematical accuracy, worthiness, visibility, appropriateness, involvement of the reader in the mathematics, the effectiveness of the presentation, the complementing of the mathematics and story, the availability of resources and mathematical information, the application of the content, and the view it presents of mathematics. Schiro’s careful, comprehensive work moved the attention to children’s literature for mathematics learning significantly forward, but the instrument he created was so thorough, it was unwieldy for practitioners. His criteria might be amenable to an ongoing project by a group of teachers within a school building, and teachers may find the literary as well as mathematical emphasis appealing, but overwhelming for individual teachers.

Following Schiro’s (1997) work, Hellwig et al. (2000) produced an instrument with five categories (accuracy, visual, and verbal appeal, connections, audience, and the “wow” factor), aimed at guiding teachers in effectively evaluating books. When applying these criteria to an evaluation, the authors warned that few books will score highly in all criteria, but that most books will have particular strengths highlighted by the instrument. Additionally, teachers are advised to keep in mind that the tool is a guide and should be used in conjunction with their judgment as professional educators. Such a tool may be best suited to teachers desiring a simple, global evaluation of a book, rather than a tool for more structured planning and decision making. While Hunsader (2004) lauded Hellwig et al. (2000) criteria for efficiency, she found their scheme lacking due to dropping Schiro’s independent evaluation of mathematical and literary quality and his criteria for assessing both the mathematical and literary appropriateness of the book.

In her book evaluation scheme Hunsader (2004) aimed to achieve a middle ground, retaining some of Schiro’s articulation but closer to Hellwig et al. (2000) efficiency. Recognizing its potential, Hunsader adapted Schiro’s (1997) instrument from the more onerous 11-item-mathematical criteria and 11-item-literary criteria, to a more streamlined six categories, eliminating what she deemed redundancies. The six mathematics-related criteria included accuracy of math content, visibility of content and effectiveness of presentation, developmental appropriateness of math content, level to which the text facilitates the involvement of the reader, if the math content compliments the narrative, and how many resources would a teacher need to collect in order to use the text successfully. For the scoring on Hunsader’s adapted model, five questions guided the evaluation of the literary quality of the book, regarding the plot’s development, the writing style, the revelance of the illustrations, the developmental appropriateness, the coherence of the components of the book, and a sense of respect for the reader. Hunsader stated her criteria clearly and the rubric would be straightforward to implement. She used it to assess 77 books, each coming from one of two primary grades mathematics curricula and found many of the books rated too low to recommend for use in mathematics learning. However, her book evaluation process drew criticism.

Carrying Hunsader’s (2004) work further, Nesmith and Cooper (2010) adopted her evaluation scheme with only a change in scoring. However, they noted the lack of clarity from Hunsader regarding how many reviewers scored books for her article, perhaps only Hunsader herself. They thus argued that the likelihood of multiple interpretations of books poses a challenge for the evaluation process. This may stem, at least in part, from fundamental differences in how mathematics concepts may be viewed within a book. As a result, they analyzed book evaluations from 30 reviewers representing mathematics professors, mathematics educators, English professors, literacy professors, and third-grade teachers. The group with the lowest agreement, while still acceptable, was the third-grade teachers. Unlike other respondents, they sometimes evaluated books given caveats for their implementation with a classroom. While this inclination may speak to the teachers’ experience in the classroom and flexible planning, it but them less in sync with the other groups of raters, raising the question of whether it can be assumed that these groups share a common approach to book selection and use in mathematics learning, and if not, for what the potential mismatches in views imply for practice and research. Nesmith and Cooper could have developed or addressed this concern more directly; it remains a significant question.

Rather than conducting their own evaluations or soliciting only classroom teachers’ reviews, van den Heuvel-Panhuizen and Elia (2012), like Nesmith and Cooper (2010), gathered and synthesized experts’ opinions about the “powerful characteristics” of picture books for mathematics education. Using this Delphi method, to draw on seven experts’ knowledge and opinions, their framework was revised and tested with three picture books. Also, notably, these researchers gave important consideration to the ideal relation between the text and illustrations in a book. van den Heuvel-Panhuizen and Elia (2012) recommended that text and illustrations should not be convey redundant information, arguing that this lack of redundancy is beneficial because, “the various and complex interactions between image and text (Nikolajeva and Scott, 2000) do not only enhance children’s attention and engagement, but also help children discover different ways of connecting words and illustrations to construct meaning, and thus extend and develop their interpretive sophistication (Wolfenbarger and Sipe, 2007)” (p. 18). Such an emphasis on construction of the book and decoding by the readers differs notably from Schiro’s (1997) emphasis on explicit mathematics being represented in words and pictures. Explicit presentations of mathematics in picture books may risk being perceived as pseudotextbooks (Nesmith and Cooper, 2010), and such books are well-represented among current tradebooks. As argued in the earlier sections on communication, representation, and connections, mathematical meanings can be recognized through the words and pictures and the experiences with them, even without explicit presentations of the mathematical concepts.

In the most recent evaluation scheme, van den Heuvel-Panhuizen and Elia (2012) drew upon the approaches mentioned previously to identify the learning-supportive characteristics of picture books for their use in supporting mathematics learning. It is clear from their results that the framework greatly assisted experts in recognizing the learning–supportive aspects of literature as compared to their recognition of such content without the use of the tool, but then the question remains of how it could be adopted by others, including teachers, more broadly. This dilemma brings us now to the processes of teacher education and professional development.

Each of the coding schemes offered a means of evaluating books to use in children’s mathematics learning and they relied on various forms of expertise to evaluate the books. None of these studies compared the rated books in their implementation with children individually or in classroom settings. Given the differences in emphases of the evaluation criteria and the different approaches to promoting learning with them, such as through teacher- or adult- scaffolded discourse, as opposed to using the books as springboards to a subsequent activity, comparisons of books and their implementations may be a greatly informative line of work.

## TEACHER EDUCATION AND ONGOING TRAINING

Addressing the ongoing concern that students are not adequately prepared to engage in mathematically rich problem solving and discourse has been a concern of pre-service teacher preparation programs and in-service teacher professional development for some time now. We now address the role of children’s literature in teachers’ mathematics-related professional development. By looking specifically into geometric knowledge attainment of students and geometric content knowledge levels of teachers, which we again note has had significant gaps, we examine one component of mathematics as a whole and highlight the ways in which teachers can improve instruction and student outcomes. The primary concerns for professional development are the inadequate training and development available to teachers along with their use of limiting curricular materials, as this combination often results in the perpetuation of geometric misconceptions in their students (Clements and Sarama, 2000). Additionally, knowledge of individual children’s developmental levels is also lacking, preventing teachers from providing instruction that is “consistent with their developmental process and individual differences” (Inan and Dogan-Temur, 2010, p. 457).

Chard et al. (2008) stated that student readiness to engage with mathematics concepts begins in infancy and must be supported through both informed, teacher-led instructional and repeated experiences with geometric content. Incorporating appropriate interventions during times of critical development of mathematical knowledge is a preventative strategy for supporting increased, accurate content knowledge in students. Likewise, Cotti and Schiro (2004) recommended that teachers identify their own ideological positions and how they influence their use of children’s literature specifically during mathematics instruction. In order to reinforce their recommendation, they have created The Mathematics and Children’s Literature Belief Inventory, which brings about deeper instructional awareness regarding “purposes, teaching, learning, knowledge, childhood, and evaluation” (p. 344). Awareness of these ideological beliefs also sparked discussion and reflection between teachers, which informed their practice. Identifying teacher’s perceptions of their areas of growth provides a possible point of entry for exploring what effective professional development opportunities must encompass in order to meet the needs of individual teachers.

Learning experiences with children’s literature are likely prone to confusion and errors as any other instructional means. Oberdorf and Taylor-Cox (1999) elucidated the ways in which some of the more common misconceptions are passed on to students through their identification of books containing such errors. They identified *The Silly Story of Goldie Locks and the Three Squares* (MacCarone, 1996) as an example of a book containing misconceptions due to the mismatch between the illustration and the text, as the shapes pictured are three solids yet they are named as two-dimensional shapes: circle, square, and triangle. Presenting such mismatches may seem trivial and easily corrected; however, the reality is that students retain these misconceptions as truths and experience confusion when trying to connect this knowledge to future learning. Without adequate experiences not only with the mathematical content, but also planning for learning with picture books, either in preparation programs or professional development teachers may not recognize such errors.

## RESPONDING WITH PROFESSIONAL DEVELOPMENT

Effective instruction with children’s literature must thus go beyond dissemination of theoretical guidelines and well-outlined narratives in practitioner focused journals to professional development for more profound change. Clements and Sarama (2011) outlined specific recommendations for accurately developing a teacher’s knowledge of early geometric concepts. Professional development of this sort is intensive and focuses not only on teaching teachers how to increase student knowledge and outcomes, but also assesses and measures the growth of the teacher’s knowledge. To begin broadly, “effective professional development ought to be extensive, ongoing, and reflective (p. 142),” meaning that schools must support teachers by providing development at multiple time points over the course of a year. During these sessions teachers are focused on specific geometric content knowledge aimed at connecting their knowledge to the understandings of their students (Ball et al., 2008). Teachers should be active participants in their development as they plan and create manipulative tools, engage in defining and discussing terminology, and examine the pedagogical implications for their students. Participation in high-quality professional development of this sort has successfully improved teachers’ geometric content knowledge as assessed by their progression through the levels of van Hiele’s (1999) model of geometric thinking.

As teachers strive to connect professional development to classroom practice, they often start by searching for supplemental materials that reinforce their newly attained knowledge. In the field of early childhood education, picture books are a familiar gateway for introducing purposeful, content-specific concepts to young children (Shatzer, 2008). When searching for such literature great care must be taken to evaluate the quality of the content when making these choices, as children’s literature is too often inaccurate and laden with misinformation (Oberdorf and Taylor-Cox, 1999) or unengaging. Teacher professional development that incorporates practicing strategies for critically evaluating geometry based literature, materials, and student content knowledge will lead to richer classroom discussion and higher quality opportunities for student learning. Specifically, for recognizing shapes and their attributes, teachers can focus on gathering literature that presents geometric shapes in a variety of colors, sizes, and orientations while also presenting non-examples to deepen student discussion (Clements and Sarama, 2000). When teachers have experiences that promote their trust in and access to high-quality, accurate literature for use in their instruction, they can pay closer attention to preventing future potential learning misconceptions in mathematics (Chard et al., 2008).

### PROFESSIONAL DEVELOPMENT FOR INDIVIDUALS

More specifically, professional development opportunities should be created and teachers should seek out professional development that meets their individual learning needs while also translating the targeted instructional practices into their own instructional techniques. At times this may require teachers to seek out instruction or information on an individual level rather than in school-wide professional development. Teachers’ efforts can be supported through the use of practitioner-based publications featuring titles like *Mathematizing Read-Alouds in Three Easy Steps* by Hintz and Smith (2013). The authors provided a way to connect math and storybooks through a three-step framework of choosing, exploring, and extending the text all in one short, peer-reviewed, accessible article. In addition to reading about instructional strategies, opportunities to read and review the content of children’s books for their quality and connectedness to mathematical topics (LeSage, 2013) can be self-guided development.

Resources such as Shatzer’s (2008) article *Picture Book Power* can be used by teachers to identify literature that has math content and organized it into specific math concepts for teachers while she also addressed how to use literature without specific math content. The author recommended that teachers focus on first leading students in enjoying the illustrations and text and then making connections to math content areas when reviewing the text. This type of modeled, purposeful instruction can ultimately become part of children’s thinking as well as part of their own personal interaction with books. As teachers use articles such as the two mentioned above, they are developing themselves professionally while also making an immediate improvement to their teaching. However, teachers’ independent professional development should be supplemental to efforts with others such as within a school or another teacher community. Collaborations among professionals can not only be effective, but may indeed be essential for developing effective planning and implementation of learning experiences with picture books, based on the strong evidence provided by Nesmith and Cooper (2010) and van den Heuvel-Panhuizen and Elia (2012).

### PROFESSIONAL DEVELOPMENT WITH GROUPS

Individual professional development cannot meet all of a teacher’s needs and may decide to seek the support of qualified instructors and or colleagues. One example that supports this thinking is the implementation of a structured set of lesson plans as outlined in the Early Learning in Mathematics Program (ELM) which increased both teachers’ confidence in teaching geometric concepts and their students’ geometric content knowledge over time (Chard et al., 2008). Picture books can then be incorporated as another source of geometric representations that need to be critically evaluated for their geometric content knowledge. As teachers deepen their pedagogical content knowledge for geometry, they will be better prepared to guide their students during discussions of the mathematics connected through texts.

A persistent challenge with short-term or limited professional development activities is the lack of support for continuing the implementation of the teachers’ learning beyond the bounds of the professional development sessions. Recently, Brendefur et al. (2013) reported the positive impact 8 h of teacher professional development had on their classrooms’ 4 year olds’ mathematical knowledge. This professional development was structured to focus on the content of four mathematical domains, number, interpreting relationships, measurement, and spatial reasoning. Teachers who participated in this professional development took with them scripted lessons to enact as centers in their classrooms. Teachers could reference scripts as a way of seeking ongoing support after the conclusion of the professional development. Such a model could be implemented for the planning and implementation of integrating children’s literature into mathematics learning, offering some reliable resources for implementing activities with picture books.

### COACHING

The coaching model of professional development supports teachers through sustained one-on-one interaction during instructional times in the classroom and during group-centered initiatives led by coaches. Poglinco and Bach (2004) examined coaching over the course of 1 year finding that complexities within the relationship between teacher and coach can be unexpected. These relational aspects can have an impact on the effectiveness of the coaching model as a tool for delivering professional development. The authors also point out the strengths coaching brings when well-trained individuals are implementing the practice. According to Keller (2007) coaching meets the needs of teachers because it embraces the design of high-quality professional development as it is sustained over time, becomes part of a teachers’ instruction, and promotes student achievement.

An example of the effectiveness of coaching is outlined by Rudd et al. (2009) as they investigated the language teachers use to supplement mathematical interactions in their research on teachers use of Math Mediated Language. Whole-group professional development was provided for teachers with the caveat that additional coaching in the classroom would be provided after the initial professional development intervention. Overall, the professional development had a positive effect on teachers’ use of math language. However, teachers did not experience the increase until the onset of a coach in their classrooms, suggesting that perhaps follow-up coaching may be key to translating professional development into practice.

Poglinco and Bach (2004) warned that although coaching has been identified as an effective professional development model, teachers and schools must be informed as to the complexities before fully adopting the model. Considering the following aspects prior to implementing a coaching model are key according to Keller (2007): how it will be funded, how to define, select, and evaluate coaches, how principals and districts will support the coaches, and lastly the details of how often coaches will work with teachers in the schools. Regarding the use of picture books in particular, coaching can offer an ongoing relationship with the coach and potentially other teachers as well, to evaluate books and then plan, implement, observe, assess, revise, and retry learning experiences with those books. Teachers can discuss with their coach and their peers which books contain accurate representations of shapes and descriptive text of those shapes, for instance, and which experiences with hands-on materials and the spatial environment of the classroom would be especially effective.

## FUTURE DIRECTIONS AND RECOMMENDATIONS

### RECOMMENDATIONS FOR TEACHERS

Whichever forms of preparation and professional development are implemented teachers must be well prepared to guide the children’s *mathematics* learning through any instructional technique, including the use of children’s literature. At least in the United States, primary grades and early childhood teachers often express a preference, interest in and greater comfort with reading and literature than mathematics, but to guide and scaffold students’ mathematical discourse and interactions with materials including books and hands-on materials, teacher must have sound content knowledge in mathematics and pedagogical content knowledge for teaching mathematics.

As well prepared as any individual teacher may be, he or she could benefit greatly from well-structured, well-focused interactions with colleagues near or far. Creating collaboratives of teachers and other experts, as Nesmith and Cooper (2010) and van den Heuvel-Panhuizen and Elia (2012) did, may not be feasible on a large scale, but especially with the advent of social media and interactive web-based technologies, possibilities may now exist that did not when using children’s literature first became popular. The use of these technologies can be a means of bringing educators together even across continents to share ideas for lessons and activities, to create collaboratives of book reviews, and to be a louder collective voice demanding publishers offer high-quality picture books, not boring, mathematically impoverished, or pseudotextbooks, for mathematics learning. The call for higher quality children’s literature for mathematics learning harkens back to Schiro (1997) and yet many low quality books may be on school shelves, identified as mathematics resources, or on booklists for suggested choices for incorporating children’s literature into mathematics learning experiences. When high-quality books have been identified, they should be shared through venues such as blogs, social media, and outreach projects to inform parents and others, not only of the existence of such high-quality literature appropriate for supporting children’s mathematical thinking, but also suggestions for reading experiences with them.

### RECOMMENDATIONS FOR RESEARCH

Almost two decades ago Hong (1996) called for more research into the use of picture books for children’s learning and this need persists. The greatest need is for well-designed empirical studies to test the effectiveness of different book types (narrative and nonnarrative; those with text and those wordless books, etc.), different means of interacting with them (e.g., to promote mathematical discourse, to introduce a hands-on activity), different methods of implementation, and different models for linking to other mathematics experiences. Edelman (2012) offered a critique of the surge in work on using children’s literature for mathematics learning and noted that many works are author testimonials or with the assumption that books will necessarily be effective for mathematics learning, rather than providing evidence for it. While practitioner-targeted guidance may support the ongoing use of books in mathematics learning, the research community must provoke issues such as characteristics of books and implementation to document the unique benefits or especially effective practices for using children’s literature for mathematics learning. Unsupported claims about books’ power must not be accepted and must not persist.

Ultimately, to continue to endorse the use of children’s literature, positive impacts on teachers and children must be documented through multiple methods of investigation, including large-scale experiment-control studies, research with teachers, and fine-grained studies of individual learners. Important and high-quality work has been done on children’s discourse during book reading (e.g., Anderson et al., 2004; van den Heuvel-Panhuizen and Elia, 2012), but significant questions remain such as measuring the children’s cognitive engagement (McLaughlin et al., 2005; van den Heuvel-Panhuizen and van den Boogaard, 2008), classroom practices, implications of learner individual differences, and due to its importance and underrepresentation, the impact of children’s literature on children’s learning of fundamental geometry concepts.

## CONCLUSION

Too often in materials for practitioners such as online resources for teachers, we see research-verified statements like “Literature can also relieve anxiety” (Whitin and Wilde, 1995, p. 9) but removed from the context, reasons, and implications that authors like Whitin and Wilde presented. For example, imagine an individual teacher coming across that statement regarding anxiety, but in isolation, and taking that statement at simple face value. Using read-aloud literature would not *necessarily* relieve anxiety for a child with hearing difficulties or children whose home language differed from the classroom. To aid teachers’ implementation of learning mathematics, including geometry, with picture books, the larger communities of mathematics educators and early childhood educators must strive to shine light on and make widely visible the processes and choices with children’s literature that can make it beneficial for student learning. In the absence of efforts on the part of not only individuals, but also the education community, children’s literature may remain yet at its hypothesized, rather than realized potential.

It is remarkable how contemporary Farr’s (1979) words sound even though they are decades old: she closed her article by saying, “We need more books based on concepts rather than abstract symbols, giving them direct application to reality and to the child’s world. And, equally important, we need books to expand the world of mathematical ideas for children beyond those they will learn in school. Children must be exposed to the power and versatility, usefulness, and flexibility of modern mathematics through one of our most important forms of communication - the printed word” (p. 104). Researchers and educators have both the opportunity and responsibility to do so.

## Conflict of Interest Statement

The authors declare that the research was conducted in the absence of any commercial or financial relationships that could be construed as a potential conflict of interest.
