# Corrigendum: Learning mathematics in two dimensions: a review and look ahead at teaching and learning early childhood mathematics with children's literature

**DOI:** 10.3389/fpsyg.2014.00854

**Published:** 2014-08-08

**Authors:** Lucia M. Flevares, Jamie R. Schiff

**Affiliations:** Department of Teaching and Learning, The Ohio State UniversityColumbus, OH, USA

**Keywords:** picture books, children's literature, early childhood, mathematics, geometry, professional development

The reference

Van den Huevel-Panhuizen, M. (2012). Mathematics education research should come more often with breaking news. Paper presented at the Freudenthal Institute for Science and Mathematics Education, Utrecht University.

should be:

Van den Heuvel-Panhuizen, M. (2012). Mathematics education research should come more often with breaking news. Lecture on the occasion of receiving the Svend Pedersen Lecture Award 2011. Stockholm: Stockholm University, Department of Mathematics and Science Education, 1 February. http://www.mnd.su.se/polopoly_fs/1.76181.1329229188!/menu/standard/file/svendPedersenLecture_120205.pdf

## Conflict of interest statement

The author declares that the research was conducted in the absence of any commercial or financial relationships that could be construed as a potential conflict of interest.

